# Natural Approaches for Neurological Disorders—The Neuroprotective Potential of *Codium tomentosum*

**DOI:** 10.3390/molecules25225478

**Published:** 2020-11-23

**Authors:** Joana Silva, Alice Martins, Celso Alves, Susete Pinteus, Helena Gaspar, Amparo Alfonso, Rui Pedrosa

**Affiliations:** 1 MARE—Marine and Environmental Sciences Centre, Polytechnic of Leiria, 2520-630 Peniche, Portugal; alice.martins@ipleiria.pt (A.M.); celso.alves@ipleiria.pt (C.A.); susete.pinteus@ipleiria.pt (S.P.); hmgaspar@fc.ul.pt (H.G.); 2 Department of Pharmacology, Faculty of Veterinary, University of Santiago de Compostela, 27002 Lugo, Spain; amparo.alfonso@usc.es; 3 BioISI—Biosystems and Integrative Sciences Institute, Faculty of Sciences, University of Lisbon, 1749-016 Lisboa, Portugal; 4 MARE—Marine and Environmental Sciences Centre, ESTM, Polytechnic of Leiria, 2520-630 Peniche, Portugal

**Keywords:** seaweed, marine natural products, neurodegenerative disease, SH-SY5Y cells, oxidative stress, mitochondrial dysfunction

## Abstract

Parkinson’s disease (PD) is the second most common neurodegenerative disorder, and is characterized by a progressive degeneration of the dopaminergic neurons in the *substantia* *nigra*. Although not completely understood, several abnormal cellular events are known to be related with PD progression, such as oxidative stress, mitochondrial dysfunction and apoptosis. Accordingly, the aim of this study was to evaluate the neuroprotective effects of *Codium tomentosum* enriched fractions in a neurotoxicity model mediated by 6-hydroxydopamine (6-OHDA) on SH-SY5Y human cells, and the disclosure of their mechanisms of action. Additionally, a preliminary chemical screening of the most promising bioactive fractions of *C. tomentosum* was carried out by GC-MS analysis. Among the tested fractions, four samples exhibited the capacity to revert the neurotoxicity induced by 6-OHDA to values higher or similar to the vitamin E (90.11 ± 3.74% of viable cells). The neuroprotective effects were mediated by the mitigation of reactive oxygen species (ROS) generation, mitochondrial dysfunctions and DNA damage, together with the reduction of Caspase-3 activity. Compounds belonging to different chemical classes, such as terpenes, alcohols, carboxylic acids, aldehydes, esters, ketones, saturated and unsaturated hydrocarbons were tentatively identified by GC-MS. The results show that *C. tomentosum* is a relevant source of neuroprotective agents, with particular interest for preventive therapeutics.

## 1. Introduction

Neurodegenerative diseases are estimated to surpass cancer as the second most common cause of death among the elderly [1]. For this reason, in recent years there has been a great deal of interest in the search for new, safer, and effective neuroprotective agents that can be used in the therapy of neurodegenerative diseases such as Parkinson’s disease (PD) [1,2]. PD is one of the most impactful human diseases without an effective cure, making imperative the research on innovative therapeutics [3]. Worldwide, there is a growing interest in the development of more selective and effective therapeutic agents to prevent or to delay the progression of neurodegeneration [4].

PD involves the loss or degeneration of dopaminergic neurons in the *substantia nigra pars compacta* (SNpc) and the accumulation of Lewy bodies, and abnormal intracellular aggregates containing proteins such as alpha-synuclein, sinfilin-1 and ubiquitin [5]. The etiology of PD remains largely unknown [6]; however, it is assumed that the main mechanisms involved in the development and progression of this disease are associated with oxidative stress, mitochondrial dysfunction, neuroinflammation, and apoptosis, among others, which act together leading to degeneration of dopaminergic neurons [7].

Natural products obtained from marine organisms have been shown to be a potential source of novel therapeutic agents. Among these organisms, macroalgae have been reported for their ability to produce relevant bioactive compounds [8], mainly derived from their secondary metabolism as a defense response to environmental variations [2]. These metabolites are known to exhibit a broad range of bioactive properties such as antioxidant [9], antimicrobial [10], anti-inflammatory [11], anti-tumoral [12], and neuroprotective [13]. Thus, these metabolites can inspire the development of new drugs for the therapy of various pathologies, such as cancer, diabetes, and neurodegenerative diseases [2]. Concerning neurodegenerative disorders, the benefits of seaweed-derived components on the prevention and/or treatment of neurodegeneration were previously reported [14]. Recently, Souza and co-workers [15] reported the neuroprotective activity of kappa-carrageenan isolated from the seaweed *Hypnea musciformis* on 6-hydroxydopamine (6-OHDA)-induced neurotoxicity on SH-SY5Y cells by modulation of the mitochondrial membrane potential and the decrease of Caspase-3 activity. Wen and co-workers [16] showed that the compound austrasulfone isolated from the marine soft coral *Cladiella australis* has potent neuroprotective effects against the 6-OHDA-induced neurotoxicity on the neuroblastoma SH-SY5Y cell line, as well as anti-inflammatory activity on RAW 264.7 macrophage cells. Additionally, studies carried out by Gao and co-workers [17] demonstrated the neuroprotective effect of the sulfated polysaccharide fucoidan on PC12 cells by blocking the H_2_O_2_-induced apoptosis, scavenging ROS formation and reducing lactate dehydrogenase activity. The authors verified that this fucoidan protected the neurocytes against H_2_O_2_-induced apoptosis via reducing ROS levels and activating PI3K/Akt signaling pathway.

In the present work, the green macroalgae *C. tomentosum,* found in the intertidal zone of the Portuguese coast [18] was evaluated for its ability to produce neuroprotective substances. The number of studies focusing on the biological potential of this species is limited, since only one report has revealed the antioxidant, anti-genotoxic, anti-tumorigenic and hypoglycemic potential of crude ethanolic extracts [18]. On the other hand, Silva and co-workers [13] demonstrated the neuroprotective capacity of *C. tomentosum* methanol and dichloromethane crude extracts, highlighting the need to pursue the investigation focused on the therapeutic potential of this species. Thus, the present work is aimed at the fractionation and identification of the bioactive molecules produced by *C. tomentosum*, the evaluation of their antioxidant activity, and the understanding of the mechanisms behind their neuroprotective effects.

## 2. Results

### 2.1. Yields, Total Phenolic Content and Antioxidant Activity of Codium tomentosum Fractions

**Figure 1:**
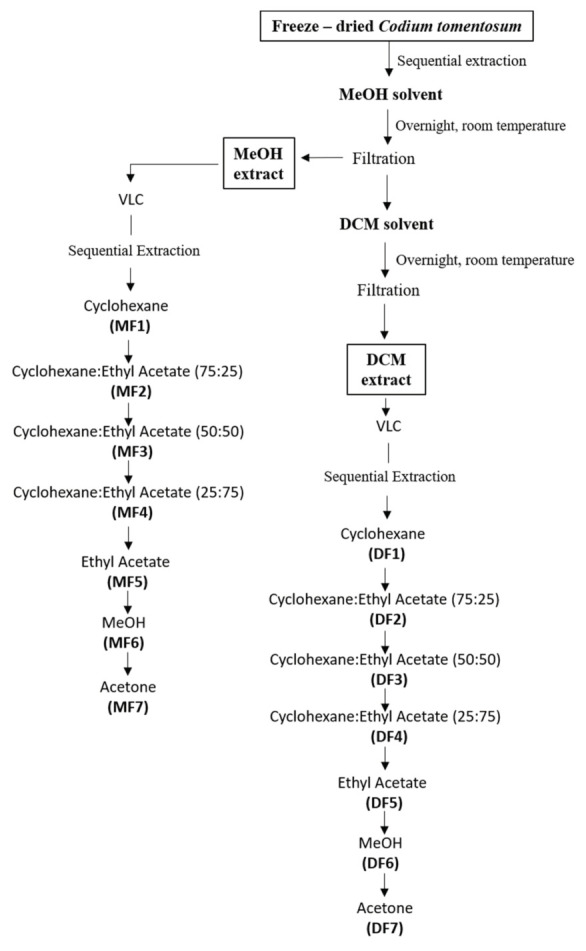
Extraction and fractionation flowchart of the green seaweed *Codium tomentosum*.

Freeze-dried samples of green seaweed *C. tomentosum* were sequentially extracted overnight with methanol (MeOH) and dichloromethane (DCM) (in a biomass/solvent ratio of 1:40) with constant stirring at room temperature. The resulting solutions were filtrated, and the organic phases were evaporated in a rotary evaporator (IKA HB10), at 30 °C. Each crude extract was fractionated by vacuum liquid chromatography (VLC) affording a total of 14 fractions (MF1-7 and DF1-7, respectively). An overview of extraction and fractionation sequences is depicted in Figure 1.

The amounts of phenolic compounds and the antioxidant activity of all these fractions were evaluated.

The fractionation yields, the total phenolic content (TPC), and antioxidant capacity assessed through 2,2-diphenyl-1-picrylhydrazyl radical (DPPH) scavenging activity, oxygen radical absorbance capacity (ORAC), and ferric reducing antioxidant power (FRAP) assays of the different fractions obtained from the green seaweed *C. tomentosum* are summarized in Table 1.

As shown in Table 1, the highest fractionation yields were achieved through the elution with methanol (DF6—13.88%; MF6—6.73%), and acetone (MF7—16.90%; DF7—8.58%), while lower yields were obtained with the less polar solvent cyclohexane (MF1—0.09%; DF1—0.57%).

Regarding the amounts of phenolic compounds, DF2 fraction from dichloromethane crude extract presented the highest TPC (10.43 ± 0.40 mg GAE/g extract) followed by MF4 fraction from methanolic crude extract (7.30 ± 0.47 GAE/g extract). On the other hand, MF1 and MF7 fractions presented the lowest TPC (2.24 ± 0.15 GAE/g extract and 2.47 ± 0.05 GAE/g extract, respectively). Concerning the antioxidant activities, none of the fractions demonstrated potential to reduce the DPPH radical. In the ORAC assay, DF7 fraction showed the highest antioxidant activity (193.85 ± 14.30 µmol TE/g extract) followed by DF6 (165.34 ± 6.42 µmol TE/g extract) and DF4 (144.15 ± 2.02 µmol TE/g extract) fractions, respectively. MF2 fraction exhibited the lowest capacity to reduce peroxyl radicals (28.92 ± 0.51 µmol TE/g extract). Fractions DF3 (825.73 ± 32.59 µM FeSO_4_/g extract), DF4 (1008.27 ± 18.18 µM FeSO_4_/g extract) and DF5 (636.21 ± 22.21 µM FeSO_4_/g extract) revealed the highest efficiency to reduce ferric ions, while the lowest activity was displayed by DF1 (24.40 ± 1.22 µM FeSO_4_/g extract).

### 2.2. Neurotoxicity and Neuroprotective Effects of Codium tomentosum Fractions on SH-SY5Y Cells

**Figure 2:**
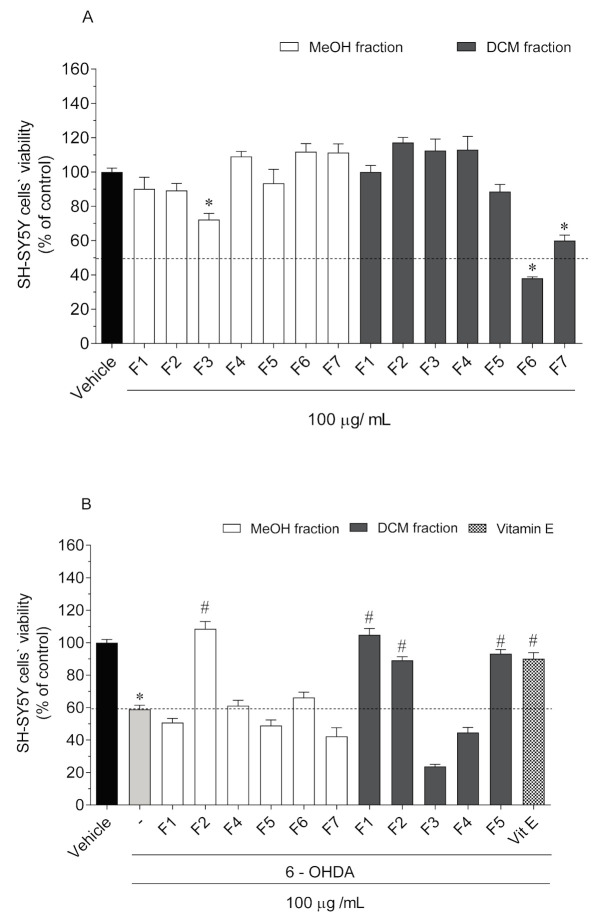
(**A**) Neurotoxicity of *Codium tomentosum* fractions (100 µg/mL, 24 h) and (**B**) neuroprotective effects of non-neurotoxic fractions (100 µg/mL, 24 h) in the presence of 6-OHDA (100 µM) on SH-SY5Y cells. (-) 6-OHDA; Vit E (Vitamin E). The values in each column represent the mean ± standard error of the mean (SEM) of three or four independent experiments. Symbols represent significant differences (ANOVA, Dunnett’s test, *p* < 0.05) when compared to: * vehicle and # 6-OHDA.

The neurotoxic effects of *C. tomentosum* fractions (100 µg/mL; 24 h) were evaluated on SH-SY5Y cells. All the non-toxic fractions were then evaluated for their neuroprotective potential on SH-SY5Y exposed to the neurotoxic effects of 6-OHDA (100 µM). Cell viability was estimated by the MTT assay and results are presented in Figure 2.

Among all fractions, only three induced a significant reduction of SH-SY5Y cells’ viability (Figure 2A): MF3 from methanolic crude extract (72.27 ± 3.56% of viable cells); DF6 (38.09 ± 2.41% of viable cells) and DF7 (60.02 ± 9.66% of viable cells) from DCM crude extract. Thus, the neuroprotective evaluation was conducted on the remaining non-toxic fractions. The exposition of SH-SY5Y cells to 6-OHDA (100 µM) for 24 h led to a reduction of cell viability of about 42% (58.92 ± 2.49% of viable cells) when compared to vehicle (100 ± 1.85% of viable cells). However, when SH-SY5Y cells were exposed to 6-OHDA, in the presence of *C. tomentosum* fractions (100 µg/mL), four samples exhibited capacity to revert (30–50%) the neurotoxicity induced by 6-OHDA to values similar to the vitamin E standard (90.11 ± 3.74% of viable cells) (Figure 2B): MF2 (108.50 ± 4.53% of viable cells); DF1 (104.8% ± 3.85% of viable cells); DF2 (89.15 ± 2.23% of viable cells); and DF5 (93.19 ± 2.53% of viable cells).

### 2.3. Neuroprotective Effects of Codium tomentosum Fractions on PD Hallmarks

**Figure 3:**
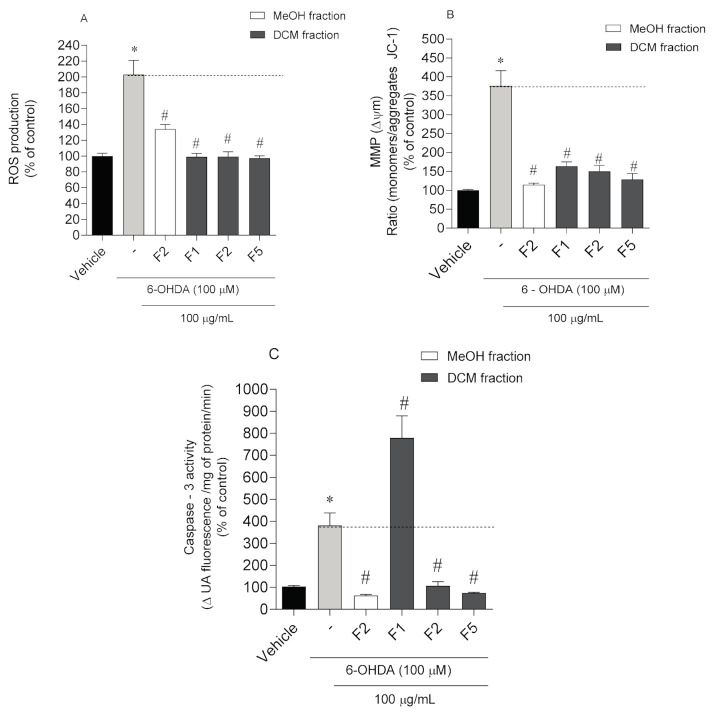
PD hallmarks evaluation on SH-SY5Y cells when exposed to 6-OHDA (100 µM) and *Codium tomentosum* fractions (100 µg/mL; 6 h). (**A**) ROS production; (**B**) changes in mitochondrial membrane potential; (**C**) Caspase-3 activity. (-) 6-OHDA. The values in each column represent the mean ± standard error of the mean (SEM) of three or four independent experiments. Symbols represent significant differences (ANOVA, Dunnett’s test, *p* < 0.05) when compared to: * vehicle and # 6-OHDA.

To investigate whether the neuroprotective effects mediated by *C. tomentosum* fractions on SH-SY5Y cells’ viability are associated with PD, several hallmarks were studied, namely oxidative stress, apoptosis, ROS production, changes in mitochondrial membrane potential, and Caspase-3 activity. The results are depicted in Figure 3.

The exposition of SH-SY5Y cells to 6-OHDA led to a marked increase of ROS levels (203.1 ± 17.78%) when compared to vehicle (100.00 ± 3.53%) (Figure 3A). In the presence of *C. tomentosum* fractions, all of them induced a significant decrease of the ROS levels being the highest reduction mediated by DF1 and DF5 fractions (101.58 ± 8.44% and 101.17 ± 4.15%, respectively).

To understand if the neuroprotective effects of *C. tomentosum* fractions were mediated by biological events on mitochondria, possible changes on MMP were studied. The exposition of SH-SY5Y cells to 6-OHDA induced a strong depolarization of the MMP (376.40 ± 39.96%) when compared with the vehicle (100.00 ± 2.05%) (Figure 3B). On the other hand, the treatment conducted with seaweed fractions exhibited a preventive effect on MMP against the depolarization induced by 6-OHDA (376.40 ± 39.96%), with the highest effect being mediated by MF2 (114.90 ± 4.04%) and DF5 fractions (128.60 ± 15.83%). Caspase-3 activity was determined to understand if the neuroprotective effects of *C. tomentosum* fractions were mediated by apoptotic pathways. The exposure of SH-SY5Y cells to 6-OHDA increased its activity (381.30 ± 57.13%) when compared with the vehicle (100.00 ± 4.83%) (Figure 3C). On the other hand, the treatment with MF2 (62.70 ± 5.59%), DF2 (107.10 ± 18.77%), and DF5 (74.43 ± 3.55%) fractions led to a marked decrease in Caspase-3 activity.

### 2.4. Chemical Characterization of Codium tomentosum Bioactive Fractions

A preliminary chemical characterization of the most bioactive fractions (MF2, DF1, DF2 and DF5) of *C. tomentosum* was performed by GC-MS. The compounds were tentatively identified by matching their mass spectra with those of reference compounds stored in the GC-MS mass spectral databases, evidencing the presence of different chemical groups such as terpenes, alcohols, carboxylic acids, esters, ketones and hydrocarbons. In fraction MF2, the presence of alkenes (1-docosene, 9-eicosene, 1-hexacosene), the fatty alcohol 1-tricosanol, and the glycol 1,2-octodecanediol was verified. Three alkanes (cyclohexadecane, eicosane, docosane), the heptafluorobutanoic acid and sebacic acid, bis (2-ethylhexyl) ester, one alcohol (2-hexyl-1-decanol), and one aldehyde (octadecanal) were detected in fraction DF1. Regarding fraction DF2, besides 2-methylundecanal and pentafluoropropionic acid, heptadecyl ester, the alcohols 2-hexyl-1-decanol and 1-tricosanol were also identified in this fraction. Finally, the monoterpenoid lactone loliolide, together with the glycol 1,2-octadecanediol, and the hexanedioic acid, bis (2-ethylhexyl) ester, were detected in fraction DF5. A terpene ketone (perhydrofarnesyl acetone) was present in fractions MF2, DF2, and DF5. Although in small amounts, these last two fractions also evidenced the presence of two peaks with characteristic mass fragmentation patterns of sterols. Additional data concerning compounds identification are available in Table S1 (Supplementary Information).

## 3. Discussion

Marine compounds have shown potential as possible therapeutic agents that can slow the processes associated with neuronal cell loss in neurodegenerative diseases, having ability to act in distinct biological targets.

Oxidative stress is believed to be one of the main triggers of neurodegenerative diseases, particularly in PD, and several studies have been carried out to understand the neuroprotective effects of antioxidant molecules [19]. These molecules have been widely discussed by several researchers as a possible therapy against neuronal death, as they have the ability to neutralize free radicals. In the present study, the antioxidant and neuroprotective potential of fractions from the green seaweed *C. tomentosum* were evaluated. Regarding their antioxidant capacity, three complementary methods (DPPH, FRAP, ORAC) were assayed and the results correlated with total phenolic content (TPC). None of the tested fractions showed significant antioxidant potential, which can be correlated with their low TPC levels. These results are in accordance with those reported by Pinteus and co-workers [9], who conducted an antioxidant screening of 27 algae collected from the Peniche coast (Portugal) in which green algae also did not show a relevant antioxidant capacity, when compared with other red and brown species. In recent years, the antioxidant potential of seaweeds has been fully documented in the literature [8,9,19], and has been attributed to the presence of a wide variety of chemical structures with high antioxidant capacity such as phenols, flavonoids, phlorotannins, sulfated polysaccharides and ascorbic acid [20].

Several mechanisms are involved in neuroprotection and, although without an expressive antioxidant ability, *C. tomentosum* fractions were evaluated for their neuroprotective potential in an in vitro model of PD. Marine extracts and derived compounds have been previously reported by several studies to exhibit neuroprotective effects against 6-OHDA neurotoxicity and, consequently, this model has been widely used to mimic experimental models of PD [8,13,20,21]. In this study, it is shown that *C. tomentosum* fractions are able to promote cells recover from the toxicity induced by 6-OHDA exposition. Our results are consistent with other studies in which *C. tomentosum* dichloromethane extracts increased neuronal cells’ viability in 35%, blocking the toxic effects of 6-OHDA [13]. Although the mechanisms responsible for inducing cell death in PD have not been totally clarified, oxidative stress is clearly involved, due to an increase of ROS, resulting in mitochondrial impairment, lipid peroxidation, DNA damage, and cell death [21,22].

Producing about 90% of cellular ROS, mitochondria have a major role in oxidative stress related diseases. In fact, mitochondrial dysfunction has long been implicated in PD pathogenesis [23]. The production of ROS by inhibition of Complex I is a key mechanism for dopaminergic (DA) neuronal damage as DA neurons are susceptible to oxidative stress due to dopamine autoxidation. The generation of ROS also induces the damage of Complex I and III and the oxidation of mitochondrial and cytoplasmic proteins, leading to their dysfunction [23,24]. Based on the results obtained in the present work, it was found that DF1, DF2 and DF5 fractions were able to prevent ROS production and mitochondrial dysfunction promoted by 6-OHDA. These results are in agreement with those previously obtained with the seaweed *Bifurcaria bifurcata* [8], in which its dichloromethane fractions prevented cell death, maintained mitochondrial function and reduced ROS production. Souza et al. [15] also demonstrated, in the same *in vitro* model, that kappa-carrageenan from the algae *Hypnea musciformis*, prevented mitochondrial potential changes and reduced H_2_O_2_ production. Additionally, several authors reported the neuroprotective properties of seaweed derived polysaccharides on 6-OHDA-induced neurotoxicity in SH-SY5Y cells [24,25,26].

Besides oxidative stress and mitochondrial dysfunction, apoptosis has also been implicated in neuronal death in PD [27]. These mechanisms are mediated by several initiators and executer caspases, via intrinsic or extrinsic pathways. The initiating caspases (-9 and -8) converge onto a common pathway of executioner caspases involving Caspase -3 and -6. The activation of executioner caspases leads to morphological features of apoptosis, such as DNA cleavage and its subsequent fragmentation. Based on the results presented here, it was verified that fractions MF2, DF2, and DF5 prevented the increase of Caspase -3 activity promoted by 6-OHDA.

A preliminary chemical screening of *C. tomentosum* most promising bioactive fractions was carried out by GC-MS analysis, and several compounds belonging to different chemical classes were identified, e.g., terpenes, fatty alcohols, carboxylic acids, aldehydes, esters, ketones, saturated and unsaturated hydrocarbons. Accordingly, Valentão et al. [18] also detected compounds belonging to some of the above described chemical classes in *C. tomentosum*, collected at Espinho coast (Portugal). The GC-MS analysis of other *Codium* species, like *C. bursa*, also suggested the presence of components belonging to the above described structural groups [26]. Numerous constituents from natural resources, namely those of marine origin having different structural features are reported for their neuroprotective properties [28,29,30,31]. Among the less polar compounds, there is great evidence that lipids play a central role in PD [32]; however, current data are still too preliminary, and more detailed studies about this issue are needed. Nevertheless, given the lipidic richness of *C. tomentosum* [18,33,34,35], the hypothesis that lipidic and other less polar constituents, including sterols [36], can be involved in the neuroprotection must not be discarded. As reported by Zhang et al. [37] the lipophilicity enhances the blood-brain barrier (BBB) penetration ability, even though BBB penetration is a highly complex process and a result of many cooperative effects. Effectively, one of the main bottlenecks associated with the development of new therapeutics for PD is the difficulty of many drugs to reach the brain, due to this highly selective semi-permeant barrier that protects brain and the spinal cord from many foreign substances. It is critical the development of deeper investigation studies in more complex in vitro models, such as co-cultures, to understand the ability of *C. tomentosum*-derived compounds to cross the BBB, and the possibility of their incorporation in smart delivery systems such as nanoparticles for brain drug delivery, tissue engineering, and new biomaterials [38]. This strategy will contribute to refine the selection of the most active compounds with neuroprotective potential suppressing the gap between in vitro and in vivo assays.

In conclusion, the current lack of studies on seaweed-derived neuroprotective agents evidences the relevance of *C. tomentosum* as a source of new compounds with potential neuroprotective properties, opening new research lines. This work showed that its bioactive fractions markedly inhibited 6-OHDA neurotoxicity induced in human neuroblastoma SH-SY5Y cells. Our results suggest that the neuroprotective effects are mediated by the mitigation of ROS generation and mitochondrial dysfunctions, together with the reduction of Caspase-3 activity. To the best of our knowledge, this is the first study focusing on the neuroprotective capacity of *C. tomentosum* in the in vitro PD model here described. Work is ongoing regarding the isolation and chemical characterization of promising constituents from the most bioactive fractions as well as compounds’ bioavailability studies on in vitro models that mimic the BBB. Nevertheless, the possibility of synergistic effects between the identified constituents need to be considered and can be regarded as an advantage, since *Codium* spp. is an edible seaweed that can be explored for nutraceutical and prophylactic purposes to overcome PD impairments.

## 4. Materials and Methods

### 4.1. Collection and Preparation of Codium tomentosum Samples

The green seaweed *Codium tomentum* Stackouse, 1797 was collected in October 2016 at Peniche coast, Portugal (39°37′05″ N, −9°38′58″ O), and transported to MARE-Polytechnic of Leiria lab facilities. The seaweed was rinsed carefully with seawater and distilled water to remove epiphytes, sand and debris. Then, it was freeze-dried (Scanvac Cool Safe, LaboGene, Lynge, Denmark), ground, and stored in a cool place protected from light, until further use.

### 4.2. Seaweed Extraction and Fractionation

Freeze-dried samples of *C. tomentosum* (445 g) were sequentially extracted with methanol (MeOH) and dichloromethane (DCM) (VWR-BDH Chemicals, Fontenay-sous-Bois, France) (in a biomass/solvent ratio of 1:40) with constant stirring, overnight. Crude dried extracts were obtained using a rotary evaporator (IKA HB10, Staufen, Germany) and/or a speed vacuum equipment (Concentrator Plus, Eppendorf, Spain). The MeOH (112.64 g, 25.31%) and DCM (6.47 g, 1.45%) crude extracts were subjected to a normal phase vacuum liquid chromatography (VLC) on silica gel 60 (0.06–0.2 mm, VWR, Leuven, Belgium). Elution was performed by using cyclohexane with increasing amounts of ethyl acetate (*v*/*v*) as the mobile phase: 1:0 (MF1, 0.02 g; DF1, 0.06 g), 2:1 (MF2, 0.66 g; DF2, 0.16 g), 2:2 (MF3, 0.18 g; DF3, 0.15 g) 1:2 (MF4, 0.11 g; DF4, 0.10 g) 0:1 (MF5, 0.13 g; DF5, 0.63 g), MeOH (MF6,1.35 g; DF6, 1.39 g), and acetone (MF7, 3.38 g; DF7 0.86 g), 400 mL of each eluent (Figure 1). Fractionation yields were determined in relation to biomass of freeze-dried seaweed. The fractions attained were subjected to a series of in vitro biological assays, in order to evaluate their antioxidant and neuroprotective potential.

### 4.3. Quantification of Total Phenolic Content (TPC)

The TPC of *C. tomentosum* fractions was determined using the Folin–Ciocalteu reagent (Sigma, Saint Louis, MO, USA) according to the method described by Singleton et al. [39] with slight modifications [8]. After 1 h of reaction in the dark, the absorbance was measured at 755 nm (Synergy H1 Multi-Mode Microplate Reader, BioTek^®^ Instruments, Winooski, VT, USA). Gallic acid was used as standard and TPC is expressed as milligrams of gallic acid equivalents per gram of dry extract (mg GAE/g of extract).

### 4.4. Evaluation of Antioxidant Activity

#### 4.4.1. 2-Diphenyl-1-picrylhydrazyl (DPPH) Radical Scavenging Activity

The ability of *C. tomentosum* fractions to scavenge the DPPH radical was determined according to the protocol described by Pinteus et al. [9]. Briefly, the reaction mixtures were incubated in the dark, for 30 min, at room temperature. The absorbance was then measured at 517 nm. The samples were tested at a maximum concentration of 100 µg/mL. A dose-response analysis (10–100 µg/mL) was performed for the samples with DPPH reduction >50% for EC_50_ determination.

#### 4.4.2. Oxygen Radical Absorbance Capacity (ORAC-Fluorescein)

The ability of seaweed fractions to neutralize peroxyl free radicals avoiding fluorescein oxidation was accomplished according to the protocol described by Dávalos et al. [40]. Samples (20 µL), and fluorescein (120 µL; 70 nM, final concentration) were added to a 96-well microplate and pre-incubated for 15 min at 37 °C. Ending this time, AAPH (2,2′-Azobis(2-methylpropionamidine) dihydrochloride (Sigma-Aldrich, Barcelona, Spain) solution (60 µL; 12 mM, final concentration) was added quickly. The microplate was immediately placed in the reader and the fluorescence (λ excitation: 458 nm; λ emission: 520 nm) recorded every minute, for 240 min. Trolox (Sigma-Aldrich, St. Louis, MO, USA) was used as an antioxidant standard. The results are expressed in µmol of Trolox equivalents/g of dry extract (µmol TE/g of extract).

#### 4.4.3. Ferric Reducing Antioxidant Power (FRAP)

The capacity of seaweed fractions to reduce F^3+^ to F^2+^ through electron donation was performed according to Benzie and Strain [41] with slight modifications [8]. FRAP reagent was prepared freshly with 0.3 M acetate buffer (pH = 3.6), 10 mM of 2,4,6-tri(2-pyridyl)-s-triazine, (TPTZ) (Alfa Aesar, Kandel, Germany) in 40 mM HCl and 20 mM ferric solution using FeCl_3_ (10:1:1) and pre-heated at 37 °C. Briefly, 2 µL of *C. tomentosum* samples were added to 198 µL of FRAP reagent and incubated at 37 °C in the dark for 30 min. FeSO_4_ was used as standard, and the absorbance was then read at 593 nm. The difference between the absorbance of test fractions and the blank reading was calculated, and results expressed as µM of FeSO_4_/g of extract (µM FeSO_4_/g of extract).

### 4.5. Evaluation of the Neuroprotective Potential of Codium tomentosum

#### 4.5.1. Cell Culture Maintenance Conditions 

The biological activities of fractions were conducted on an in vitro cellular model of human neuroblastoma (SH-SY5Y cells, strain number ACC 209) previously acquired from DSMZ (Deutsche Sammlung von Mikroorganismen und Zellkulturen GmbH) biobank. The cells were cultured according to the supplier’s instructions. SH-SY5Y cells grew in DMEM:F12 medium (Biowest, Riverside, MO, USA) supplemented with 10% (*v*/*v*) fetal bovine serum (Biowest, Riverside, MO, USA) and 1% of antibiotic mixture constituted by 1% penicillin/streptomycin (Sigma, Rehovot, Israel). SH-SY5Y cells were maintained in a humidified atmosphere with 5% CO_2_ at 37 °C.

#### 4.5.2. Neurotoxic and Neuroprotective Potential

The neurotoxicity was evaluated on SH-SY5Y cells at 75–85% of total confluence. Cells were treated with seaweed fractions (100 μg/mL) for 24 h to determine and select the non-neurotoxic samples/concentrations for the next assays. Regarding the neuroprotective assay, SH-SY5Y cells were exposed to 6-OHDA (100 µM) (Sigma, St. Louis, MO, USA) in the presence/absence of non-neurotoxic seaweed fractions (100 µg/mL). The effects on SH-SY5Y cells’ viability was then estimated by the MTT (3-(4,5-dimethyl-2-thiazolyl)-2,5-diphenyl-2H-tetrazolium bromide) assay (VWR, Solon, OH, USA). Cells were incubated with MTT (1.2 mM) for 1 h at 37 °C. Ending this time, 100 µL of dimethyl sulfoxide (DMSO) were added, and the absorbance read at 570 nm (Synergy H1 Multi-Mode Microplate Reader, BioTek^®^ Instruments, Winooski, VT, USA). The results were expressed as percentage of the control.

#### 4.5.3. Neuroprotective Effects on PD Biomarkers

##### Determination of Intracellular Reactive Oxygen Species (ROS) Levels

The ROS levels were determined using the oxidation sensitive fluoroprobe 5(6)-carboxy-2′,7′-dichlorofluorescein diacetate (carboxy-H2DCFDA) (Invitrogen, Bleiswijk, Netherlands) according to Ouazia et al. [42] with slight modifications. Briefly, SH-SY5Y cells were exposed to 6-OHDA (100 μM) in the presence/absence of seaweed fractions (100 μg/mL) for 6 h. Cells were then washed with phosphate buffered saline (PBS) and incubated with carboxy-H2DCFDA (20 μM) probe, previously dissolved in serum-free medium for 1 h at 37 °C. Ending this time, the fluorescence was read (λ excitation: 527 nm; λ emission: 495 nm).

##### Mitochondrial Membrane Potential (MMP)

Analysis of MMP was performed using the JC-1 fluorescent dye (Molecular Probes, Eugene, OR, USA) according to Silva et al. [8]. SH-SY5 cells were exposed to 6-OHDA (100 μM) in the presence/absence of seaweed fractions (100 μg/mL) for 6 h. Cells were then washed with PBS and incubated with JC-1 solution (3 µM) in the dark, for 15 min, at 37 °C. After this time, the JC-1 dye was removed, and PBS was added to wells. The fluorescence was read at 530 nm (monomers) and at 590 nm (aggregates) and 490 nm emission/excitation wavelengths, respectively. The results were calculated from the ratio between JC-1 monomers and aggregates and are expressed in percentage of control.

##### Caspase-3 Activity

Caspase-3 activity was evaluated using the caspase-3 fluorometric assay kit (CASP3F-KIT, Sigma, St. Louis, MO, USA) according to the manufacturers´ instructions. Briefly, SH-SY5Y cells were treated with 6-OHDA (100 μM) in the presence/absence of seaweed fractions (100 μg/mL) for 6 h. Ending treatment time, cells were washed with PBS, harvested, and enzyme activity determined by reading the fluorescence at 360 nm and 460 nm emission and excitation wavelengths, respectively. The results were calculated from the slope of the curve and are expressed in percentage of control.

### 4.6. Chemical Characterization of Codium tomentosum Bioactive Samples

#### Gas Chromatography-Mass Spectrometry (GC-MS) Analysis

GC–MS qualitative analysis was performed in a Shimadzu QP2010-Plus GC/MS system equipped with a TRB5MS (30 m × 0.25 mm i.d. × 0.25 µm film thickness) capillary column (Teknokroma, Barcelona, Spain) operating in the linear velocity mode. The carrier gas was helium 5.0 (Linde, Lisboa, Portugal), with a constant flow of 1 mL/min. Samples were dissolved in dichloromethane and automatically injected. Injections were performed in split mode, with a ratio of 1/9. The injector port was heated to 280 °C. The initial column temperature of 60 °C was held for 2 min, followed by a temperature ramp of 30 °C/min to 300 °C held for 15 min. All mass spectra were acquired in electron impact (EI) mode at 70 eV. The operate temperatures were 200 °C for MS ion source, and 250 °C for the liner interface. Analysis were performed in full scan mode with mass ranging from 10 to 800 *m*/*z*. Compounds were identified by matching the mass fragmentation patterns with those stored in the GC-MS mass spectral databases (Wiley 229 and NIST-National Institute of Standards and Technology libraries).

### 4.7. Data and Statistical Analysis

When applicable, results are presented as mean ± standard error of the mean (SEM). The determination of EC_50_ was attained from sigmoidal dose–response variable-slope curves using the GraphPad Prism v.8 software (GraphPad Software Inc., San Diego, CA, USA). One-way analysis of variance (ANOVA) with Dunnett’s multiple comparison of group means was employed to determine significant differences relatively to the control treatment. All other post hoc analyses were conducted using Tukey’s test. All data were checked for normality and homoscedasticity. Comparisons concerning variables, which did not meet variance or distributional assumptions, were carried out with Kruskal–Wallis non-parametric tests. At least three independent experiments were carried out in triplicate.

## Figures and Tables

**Table 1:** Fractionation yields, TPC, and antioxidant capacity of *Codium tomentosum* fractions.

Crude Extract	Fraction	Yield (%)	TPC ^a^	DPPH ^b^	FRAP ^c^	ORAC ^d^
**MeOH**	MF1	0.09	2.24 ± 0.15	>100	282.87 ± 14.27	99.91 ± 0.57
	MF2	3.29	3.96 ± 0.26	>100	288.40 ± 11.42	28.92 ± 0.51
	MF3	0.88	2.58 ± 0.13	>100	282.99 ± 2.51	52.60 ± 1.28
	MF4	0.57	7.30 ± 0.47	>100	271.33 ± 7.34	74.76 ± 0.48
	MF5	0.65	4.32 ± 0.30	>100	273.01 ± 1.81	66.32 ± 0.95
	MF6	6.73	4.38 ± 0.12	>100	292.73 ± 3.73	72.73 ± 1.36
	MF7	16.89	2.47 ± 0.05	>100	23.79 ± 8.45	98.55 ± 2.28
**CH_2_Cl_2_**	DF1	0.58	4.18 ± 0.13	>100	24.40 ± 1.22	38.88 ± 0.73
	DF2	1.52	10.43 ± 0.40	>100	67.67 ± 8.05	62.06 ± 1.67
	DF3	1.53	5.51 ± 0.75	>100	825.73 ± 32.59	98.84 ± 6.00
	DF4	1.00	3.51 ± 0.20	>100	1008.27 ± 18.18	144.15 ± 2.02
	DF5	6.35	4.30 ± 0.23	>100	636.21 ± 22.21	94.56 ± 1.02
	DF6	13.88	4.57 ± 0.17	>100	356.95 ± 3.73	165.34 ± 6.42
	DF7	8.58	5.69 ± 0.16	>100	582.11 ± 20.81	193.85 ± 14.30
	BHT	-	-	>100	2821.50 ± 51.27	142.87 ± 8.71

^a^ mg gallic acid equivalents (GAE)/g extract; ^b^ radical scavenging activity (EC_50_ µg/mL); ^c^ µM FeSO_4_/g extract; ^d^ µmol trolox equivalents (TE)/g extract. EC_50_ values were determined for a 95% confidence interval. MeOH—methanol; CH_2_Cl_2_—dichloromethane.

## Supplementary Materials

The following are available online, Table S1: Identification of compounds in *Codium tomentosum* bioactive fractions by GC-MS.
