# Supplementary material for: Natural Approaches for Neurological Disorders—The Neuroprotective Potential of Codium tomentosum
Source: Molecules. 2020 Nov 23;25(22):5478. doi: 10.3390/molecules25225478 (PMC7700523; doi:10.3390/molecules25225478)
Supplement: Supplementary file 1 [file molecules-25-05478-s001.pdf]

Table S1. Identification of compounds in *Codium tomentosum* bioactive fractions by GC-MS *

Fraction	RT (min)	Compound	MW
**MF2**	8.67	1 – Docosene	308
	8.89	Perhydrofarnesyl Acetone	268
	9.40	1-Tricosanol	340
	9.45	9-Eicosene	280
	10.08	1,2-Octodecanediol	286
	10.64	Cyclotetracosane	336
	11.34	Mono (2-ethylhexyl) phthalate	278
	13.45	1-Hexacosene	364
**DF1**	7.89	2-Hexyl-1-decanol	242
	8.33	Eicosane	282
	8.70	Cyclohexadecane	224
	10.06	Docosane	310
	10.67	Heptafluorobutanoic acid	452
	11.28	Octadecanal	268
	12.34	Sebacic acid, bis(2-ethylhexyl) ester	426
**DF2**	8.31	2-Methylundecanal	184
	8.85	Perhydrofarnesyl Acetone	268
	9.39	Pentafluoropropionic acid, heptadecyl ester	402
	10.03	2-Hexyl-1-decanol	242
	11.06	1-Tricosanol	340
	11.26	Di- *n*- octyl phthalate	390
**DF5**	8.75	Loliolide	196
	8.92	Perhydrofarnesyl Acetone	268
	10.73	Hexanedioic acid, bis(2-ethylhexyl) ester	370
	11.23	Di-*n*-octyl phthalate	390
	11.78	1,2-Octadecanediol	286

*Compounds were tentatively identified by matching their mass fragmentation patterns with those stored in the GC-MS mass spectral databases (Wiley 229 and NIST-National Institute of Standards and Technology libraries).
